# Menzerath’s Law in the Syntax of Languages Compared with Random Sentences

**DOI:** 10.3390/e23060661

**Published:** 2021-05-25

**Authors:** Kumiko Tanaka-Ishii

**Affiliations:** Research Center for Advanced Technology, The University of Tokyo, Tokyo 153-8904, Japan; kumiko@cl.rcast.u-tokyo.ac.jp

**Keywords:** Menzerath law, complexity, natural language, syntax

## Abstract

The Menzerath law is considered to show an aspect of the complexity underlying natural language. This law suggests that, for a linguistic unit, the size (*y*) of a linguistic construct decreases as the number (*x*) of constructs in the unit increases. This article investigates this property syntactically, with *x* as the number of constituents modifying the main predicate of a sentence and *y* as the size of those constituents in terms of the number of words. Following previous articles that demonstrated that the Menzerath property held for dependency corpora, such as in Czech and Ukrainian, this article first examines how well the property applies across languages by using the entire Universal Dependency dataset ver. 2.3, including 76 languages over 129 corpora and the Penn Treebank (PTB). The results show that the law holds reasonably well for x>2. Then, for comparison, the property is investigated with syntactically randomized sentences generated from the PTB. These results show that the property is almost reproducible even from simple random data. Further analysis of the property highlights more detailed characteristics of natural language.

## 1. Introduction

The theme of this article is the *Menzerath* law of syntactic structure, which has been considered to demonstrate some of the complexity underlying natural language. Originally, Menzerath observed a negative correlation between the number of phonetic constructs in a word (*x*) and the length of its constituents (*y*) [1,2,3]. An older report [4] observed the duration of the vowel /a/ in words and found that the sound is consistently shorter in longer words. Because Menzerath was the first to extensively study and publish regarding this phenomenon, recent literature has attributed the property to him, and this article follows this convention. One method to study the reasons for such a phenomenon is to formulate it mathematically. The first such functional formulation was proposed by Altmann [5], and thus, the property is often called the Menzerath–Altmann law.

Since those origins, abundant reports on this property have shown how it holds for various linguistic phenomena outside the original setting. Krott [6] showed such a relation between the word length and morpheme length. Alekseev [7] showed a relation between graphemes and the number of syllables in Russian. The original approach of phonetic investigation has been extended to actual speech [8,9,10].

For Japanese characters, Prun [11] demonstrated that the mean number of strokes per grapheme decreased with respect to the number of graphemes per kanji character. The applications extend even beyond language, as the Menzerath law has been investigated for gelada (a kind of monkey) calls [12], music [13], and even genomes [14,15]. There have also been theoretical arguments, which have involved searching for other mathematical formulations [16,17] and studying the law’s relation to other power laws of language [8,18,19], as well as information theory [20].

Recently, there have been indications that the Menzerath property also holds for syntactic structures in language [21,22,23,24]. Those authors suggested measuring the mean size of the main constituents of a sentence (*y*) with respect to the number of main constituents (*x*). The papers showed how the Menzerath property held for the authors’ respective mother tongues. However, it is unknown how well their findings apply across other languages. Hence, by using large-scale syntactically annotated corpora from the Universal Dependency dataset (ver. 2.3, with 76 languages across 129 corpora) and the Penn Treebank (PTB), this work describes a large-scale investigation of the property across languages to elucidate its universality.

The previous syntactic works mentioned above focused on showing how the Menzerath property held for specific kinds of language or language-related data. The overall understanding of the reason for the Menzerath property thus far has been centered in relation to phonetics [8,12]. For syntax, analysis has been limited to Dȩbowski [20], whose approach used the notion of grammar but concerned information theory rather than natural language syntax. In contrast, this article applies a new idea of using random dependency sentences generated from the PTB.

Such randomized analysis enables consideration of how natural language text differs from random data. The findings show that the Menzerath law is almost reproducible even from simple random data. At the same time, the detailed analysis clearly shows some aspects of natural language that are different from those of random data, which suggests that further study of the Menzerath property could lead to a better understanding of natural language.

## 2. Formulation of the Property

In this article, the term *Menzerath law* indicates the *property* whereby the size of the constituents, *y*, decreases when the total number of constituents, *x*, becomes large. Although it is a *property* of language without a solid theoretical background, it is called a *law* by convention, because it applies ubiquitously.

As mentioned above, the property was mathematically formulated by [5] in terms of three formulas, of which the most conventional is a power function,

(1) $$y\propto x^{-D},$$

or the following function,

(2) $$y\propto x^{-B}\exp\left(Cx\right).$$

When modeling Menzerath phenomena by use of these functions, the law is often called the *Menzerath–Altmann* law, as was also mentioned above.

Unlike other kinds of studies related to power laws of language, such as those on Zipf’s law [25], the mathematical formulation of the Menzerath property presents two common, basic problems of analysis when applied to certain data. First and foremost is that the number of data points is typically very small within a narrow range of a scale. When a property concerns a power distribution, it is desirable to consider at least several decades of data points [26]. This is not possible, however, for the Menzerath property: usually, the number of data points is, at most, distributed up to around 10. The second problem is that, for every point, the variation is usually very large. As a result, only the mean value exhibits a tendency to drop.

The Menzerath kind of property does exist, however, and there is interest in clarifying what exactly it is. Hence, returning to the original point conceptualized by Menzerath, this article studies the Menzerath law by defining it as follows:

The Menzerath law holds when the average of *y* tends to drop monotonically with respect to *x* for $x_{min}\leq x\leq x_{max}$,

where $x_{min}$ and $x_{max}$ depend on the corpus. What function is appropriate is not the main point of this article, but the functional parameters *D*, *B*, and *C* are nevertheless estimated to quantify the degree of decrease of *y*. This serves to provide reference to the history of the main functional argument about the property, and furthermore, the estimated values help with rough examination of the degree of decrease. The fitting is performed by the least-squares method within $x_{min}\leq x\leq x_{max}$. The error is given as the standard deviation from the fit function.

## 3. Dependency Structure

**Figure 1:**
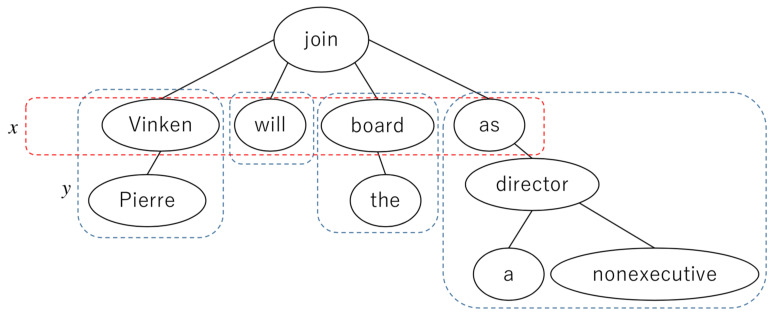
Dependency structure showing the syntactic structure of the words in a part of a sentence from the Penn Treebank (PTB [28]). In this article, *x* is the number of words in the red box, whereas *y* is the number of words in each blue box.

As known in linguistics, a dependency structure is a syntactic structure of a sentence that is described through every word of the sentence that modifies another word [27]. For example, consider the following sentence: *Pierre Vinken will join the board as a nonexecutive director.* This is a part of a longer sentence appearing in the Penn Treebank [28], which is shortened here for the purpose of explanation. The punctuation is removed for the sake of clarity, and the auxiliary verb *will* is considered here to modify the verb *join*. Figure 1 shows the dependency structure of this sentence for words. The word *Pierre* modifies *Vinken*, *Vinken* modifies the verb *join*, and so on.

Typically, the head word of a sentence is a predicate. In the community that constructs dependency-annotated corpora, there are debates on structure definitions, such as the direction of dependency relations or how to treat function words [27]. The design of dependency structures is beyond the scope of this article. Because these relations are defined and annotated consistently within a given corpus, this article follows the definitions provided in each annotation community.

Regarding the Menzerath property for syntactic structures, previous works [21,24] suggested calculating the following two values:

*x*: The number of modifiers of a word *w*.
*y*: The count of the number of words in a constituent with a head that modifies *w*.

For example, let *w* be the sentence predicate *join*. In Figure 1, *x* is the number of constituents in the red box, whereas *y* is the count of words in each blue box. Thus, four constituents each modify *join*, and they are headed by the words *Vinken*, *will*, *board*, and *as*. Thus, the number of constituents is x=4, the sizes of the constituents are y=2,1,2,4, and the mean value is y=9/4. Such counting may recursively consider heads that exist in constituents.

Following previous works [21,22,23,24], in this work, too, the main predicates of sentences were used to acquire statistics; *y* is the mean size of the constituents modifying the main predicate and *x* is the number of modifiers of the main predicate.

To calculate *y* values, the previous works took the mean of the constituent size for every sentence and as a result, they reported a smaller standard deviation for *y*, because they calculated the mean first and then the standard deviation. In this work, therefore, *y* is acquired for all constituents in a corpus for *x* of the same size, and the mean and standard deviation are then calculated for the entire corpus.

## 4. Menzerath Property of Syntactically Annotated Data

To verify how well such previous findings apply across languages, this section examines the Menzerath property for two datasets, the Universal Dependency dataset ver. 2.3 [29] and the PTB [28].

### 4.1. Universal Dependency Dataset

Results were obtained from all data in the Universal Dependency dataset ver. 2.3. Each corpus included a set of word sequences, most of which were sentences. Previously, Mačutek et al. [21] filtered the records so that the corpora included only complete sentences. Unlike in that work, the statistics were acquired here without filtering any records. This difference is an important point to which we will return in Section 5.

Table 1 lists the detailed statistics for all the experimental results appearing in this article. The first three columns contain the sentence length populations. Most sentences were much longer than 10 words, which is almost the maximum of the range of *x*, as we will see.

The 4th and 5th columns list the overall statistics characterizing the nature of the data. In the 4th column, the decrease ratio indicates the average proportion of data points among all points in the range of 1<x≤16 for which the mean constituent size of *x* decreased as compared with that of x−1. Looking at this 4th column vertically shows that around 70% to 80% of the data points in the Universal Dependency dataset had a decreasing tendency. Usually, points tend not to monotonically decrease at the head (x≤2) or tail (typically x≥10). Therefore, this proportion of 70% to 80% suggests that the Menzerath law was fairly well followed within the main middle part of the data.

The 5th column lists the average standard deviation of the data points. The values indicate that the variation of *y* was large. The remaining columns list the results of the functional fits. The standard deviation in the 5th column has a relation to the fit errors of the two functions. In particular, the errors for the function of Formula (2) (7th column) show that the fits were not necessarily good even though it has two parameters, whereas Formula (1) has one. Given that the number of data points is so small, the rest of the article uses the power function of Formula (1) as a reference.

**Figure 2:**
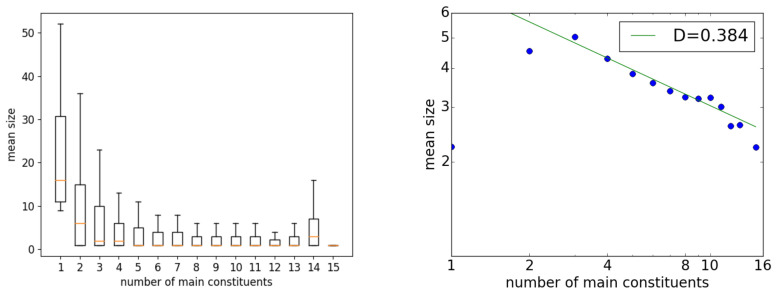
The Menzerath property for all sentences of the Czech-PDT corpus in the Universal Dependency dataset ver. 2.3: (**left**) a box plot on normal axes, and (**right**) an averaged plot on double-logarithmic axes.

The first to third rows in Table 1 list individual data for the three largest corpora of the Universal Dependency dataset. The largest is the Czech-PDT corpus (87,913 sentences), and Figure 2 shows the resulting relation between *x* and *y* for this corpus. The left graph is a box plot on normal axes, in which each box ranges between the quantiles, with the middle line indicating the median and the whiskers indicating the maximum and minimum. The right graph shows the results plotted on double-logarithmic axes, as the plot was fitted to a power function.

The data points for x=1,2 did not follow the Menzerath property, but for x>2, the data did tend to follow it. The *y* values appeared to start fluctuating at around x=10, which is natural because sentences with more than 10 main constituents are rarer than sentences with fewer main constituents. In the right graph, the points between 2<x≤10 are aligned relatively straight, and the fit to a power function gave D=0.38.

The results presented here differ from those obtained for the Czech-PDT corpus in Mačutek et al. [21], which reported a value of D=0.62 and found that the case of x=2 followed the Menzerath property. As mentioned at the beginning of this section, the main cause of this difference is that Mačutek et al. [21] carefully preprocessed the data; this issue is discussed further in Section 5.

The second largest corpus in the Universal Dependency dataset is in Russian (SynTagRus, 61,889 sentences) and had D=0.19, while the third largest is in Japanese (BCCWJ, 57,109 sentences) and had D=0.41. For each of these corpora, the values for x=1 were smaller; however, for x>1, the points decreased monotonically up to around x=10.

The fourth row in Table 1 lists the average results for the 129 corpora in the Universal Dependency dataset. Compared with the large corpora described above, the majority of the corpora contain less than 10,000 sentences and have a larger number of shorter sentences. The decrease ratio was 0.76, thus showing a monotonic decrease only within the main middle part of the data, similarly to the Czech case.

**Figure 3:**
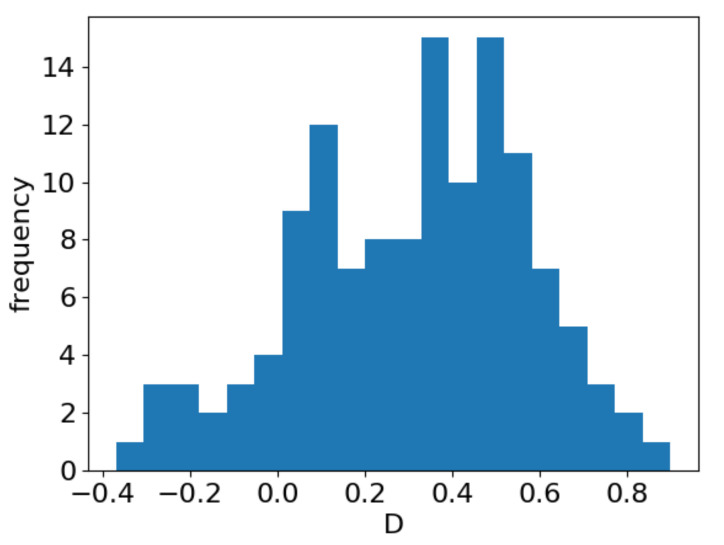
Histogram of the power exponent *D* for all 129 corpora in the Universal Dependency dataset.

Figure 3 shows a histogram of the *D* values for all 129 corpora in 76 languages. Despite some previous interest in the behavior of *D* (including its possible universality), the *D* values for syntactic structure varied and apparently depended on the corpus and also on the settings, as exemplified by *D* for the Czech-PDT corpus differing from that in a previous report [21]. A small number of corpora had negative *D* values, indicating that the data points did not present any decreasing tendency. Those corpora, however, contained only small numbers of sentences, with many short sentences, and they were often in minor languages. Overall, the Menzerath law as defined in Section 2 (i.e., the monotonic decay of *y* w.r.t. *x*) holds almost universally; the degree of decrease, however, as measured by *D* as one possibility, depends on the corpus and the settings, and it is therefore unlikely to have a narrow distributional range.

### 4.2. Penn Treebank

The same analysis was applied to the Penn Treebank (PTB) [28]—the most standard syntactically annotated dataset. The PTB is annotated using a different syntactic representation framework, namely, a context-free grammar (CFG) [30]. A CFG and a dependency structure are known to be related; however, the specific focus of each framework is different. Conversion from a CFG to a dependency structure is easier than the opposite case [31,32], and tools are available for this conversion. Accordingly, this work used chunklink, developed by Buchholz [33], to convert the PTB to a dependency structure.

**Figure 4:**
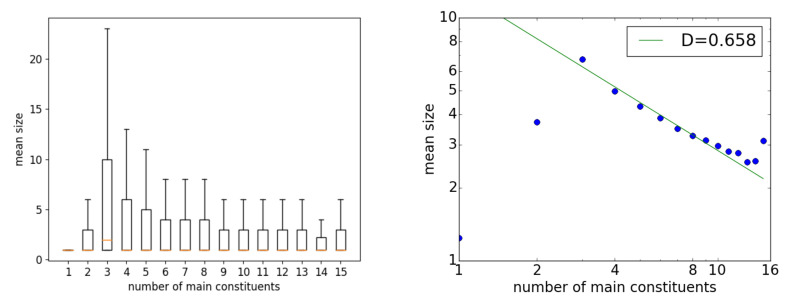
The Menzerath property for the PTB: (**left**) a box plot on normal axes, and (**right**) an averaged plot on double-logarithmic axes.

Figure 4 shows the Menzerath property results for the dependency-converted PTB. The property did not hold for x=1 or 2. For x>2, however, the points decreased and exhibited a power-law-like behavior until about x≤10 with D=0.66, which was slightly larger than the values for many corpora of the Universal Dependency dataset. The overall statistics for the PTB are listed in the fifth row of Table 1. The larger *D* was due to the average sentence lengths being longer, as will be considered in the next section.

## 5. Menzerath Property of Random Sentences

The experimental results thus far show the global reality of the Menzerath property for syntactic structures in natural language. Although the number of data points has been small, there is a common tendency of decreasing *y* with respect to *x* within the main range of the data points, and it is worthwhile to conjecture why this is so.

Previously, many papers on the Menzerath property focused on showing it with natural language or real linguistic data, but it has barely been applied to other well-defined sequences. One exception was by Dȩbowski [20], who considered the smallest grammar acquired from a text, but his use of *grammar* was information theoretic and not syntactic in the linguistic sense. Here, the statistical mechanics underlying the Menzerath property are studied by applying them to syntactically random sentences.

For a syntactic structure, if clauses are randomly independent of one another, then the size of *y* should not depend on *x*. In other words, if a sentence is generated in a truly context-free manner, then it should have D=0 across *x*. In reality, human text is not context free. Thus, by starting from a sequence that reproduces this characteristic, we will be able to consider the conditions for a corpus to reproduce the monotonic decrease of *y* with respect to *x*.

### 5.1. Generation of Random Dependency Sentences

To this end, random dependency sentences were generated stochastically by using an annotated corpus. Among various options for generation, the following procedure is one natural way to produce random dependency sentences.

1. A dependency grammar was built for the dependency-converted PTB by examining every word and recording what other words modified it. As a word *w* can be modified by the words before or after itself, the sets of modifying words *before* and *after w* were both collected with their frequency counts, and this was done for all *w*. The resulting sets of modifier–modified relations were denoted as Gb and Ga, respectively.
2. All main predicate words were collected from the corpus, and the resulting set was denoted as *H*.
3. A main predicate was sampled as w∈H according to its frequency. Then, *a sentence* was randomly generated by recursively using a function *F*, starting with the main predicate *w* as the target word. For every target word *w*, by using Gb and Ga, *F* generates modifiers with a mean length of *w*, in proportion to the frequency of modifiers. For every modifier generated in this way as a target, function *F* is called recursively. This procedure stops recursive generation when *w* without any modifier is produced, and it thus generates one sentence.
4. Step 3 was repeated 100,000 times to generate a sample corpus.
5. Step 4 was repeated 10 times to generate 10 different sample corpora.

### 5.2. Empirical Menzerath Property of Random Sentences

**Figure 5:**
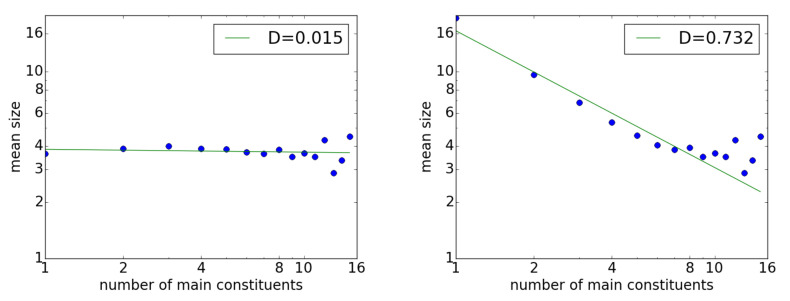
The Menzerath property for randomized sentences. The **left** graph shows the results for an entire sample corpus, whereas the **right** graph shows the results only for sentences of length ≥ 10 words.

For the 10 generated sample corpora, each containing 100,000 random sentences, the same analysis was applied as for the Universal Dependency and PTB datasets. The left side of Figure 5 shows the resulting Menzerath property graph for one such sample corpus. Following the theory, the sizes of the constituents of a random dependency structure were independent, with D=0.015 and no decreasing tendency. The average statistics of the 10 sample corpora are listed in the second row from the bottom in Table 1. The overall average was D=0.00 with a very small standard deviation.

Given that the theory was confirmed and D=0 was reproduced, the next issue is to find a condition to reproduce the Menzerath property. One way is simply by filtering out shorter sentences of length less than $N_{min}$. Any threshold $N_{min}$ would have the same effect, but following the previous section’s evidence that x=10 represents some limit, the results for $N_{min}=10$ are shown on the right side of Figure 5. Here, as the points almost showed monotonic decay starting from x=1 up to $N_{min}$, the fitting to the power function was performed between $x_{min}=1\leq x\leq 10=x_{max}$. The resulting graph shows that the Menzerath property held with D=0.732. The average statistics for this case of $N_{min}=10$ are listed in the last row of Table 1. With increasing $N_{min}$, *D* would increase gradually and could take any arbitrary value, even above 1.0.

The use of the threshold $N_{min}$ partly explains the results reported previously for real corpora. Section 4.1 noted the different results for Czech reported in Mačutek et al. [21], in which the authors preprocessed the PDT corpus to achieve better results; this was a reasonable, thoughtful procedure for obtaining a better estimate of *D*, as their goal was to investigate the Menzerath–Altmann law for Czech sentences.

**Figure 6:**
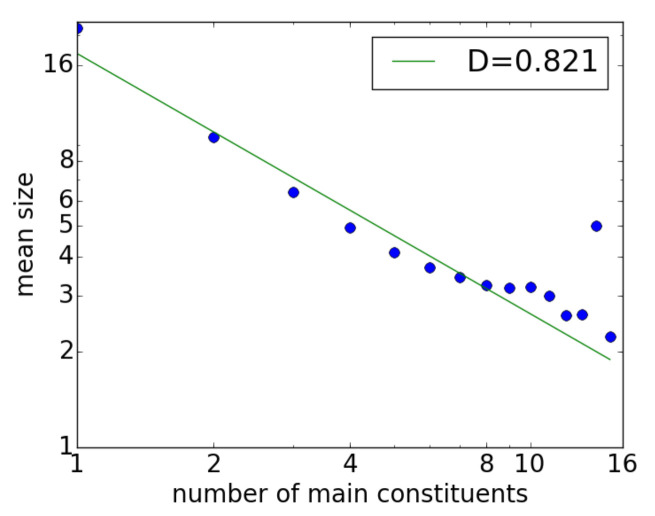
The Menzerath property for Czech-PDT corpus sentences with a length of at least 10 words.

In contrast, in the present article, the entire Universal Dependency dataset was used without any filtering, and the corpora thus contained a certain number of shorter non-sentences; as a result, the *D* value was 0.321, as mentioned in Section 4.1. By filtering out sentences shorter than $N_{min}=10$ from the Czech-PDT corpus, however, the right graph in Figure 2 becomes Figure 6, which shows the data following the power law better and the Menzerath property even holding for x=1,2. In this case, D=0.821, which is much larger than the previous value of D=0.321. Therefore, depending on the threshold for the sentence length, the exponent *D* can take an arbitrary value.

More generally, for the natural language results in Table 1, *D* became larger when the mean of a sentence was longer. For example, *D* was larger for the PTB because the mean sentence length was indeed larger than in the other corpora. This suggests the possibility that the effect of the distribution of the sentence lengths to be biased toward a longer regime is partly the cause of the sharper decrease in the Menzerath property.

### 5.3. Analytical Rationale

The analytical rationale of the previous section is given as follows. Let u(n,x) be the number of sentences of length *n* whose number of main constituents is *x*. Then, *y* as a function of *x* is obtained as follows:

(3) $$\begin{array}{ccc} y(x) & = & \frac{1}{\sum_{n\geq x}u\left(n,x\right)}\sum_{n\geq x}\frac{n}{x}u\left(n,x\right)\\ & = & \frac{1}{x}\frac{\sum_{n\geq x}nu\left(n,x\right)}{\sum_{n\geq x}u\left(n,x\right)}. \end{array}$$

Note that the summation is taken across *n* for a given *x*. In the first row, for a sentence of length *n*, the constituent size is n/x, and there are u(n,x) such sentences. Therefore, the *y* value is its total sum divided by the total number of sentences, the sum of the u(n,x). The second row factors out the term 1/x, which is not dependent on *n*. Hence, the second term shows the ratio of different moments of u(n,x), integrated for n≥x, and it therefore depends on the value of *x*. Whether the Menzerath property holds for *y* thus lies in the nature of this distribution u(n,x). Hereafter, let *v* denote the second term, i.e., $v\equiv \left(\sum_{n\geq x}nu\left(n,x\right)\right)/\left(\sum_{n\geq x}u\left(n,x\right)\right)$.

**Figure 7:**
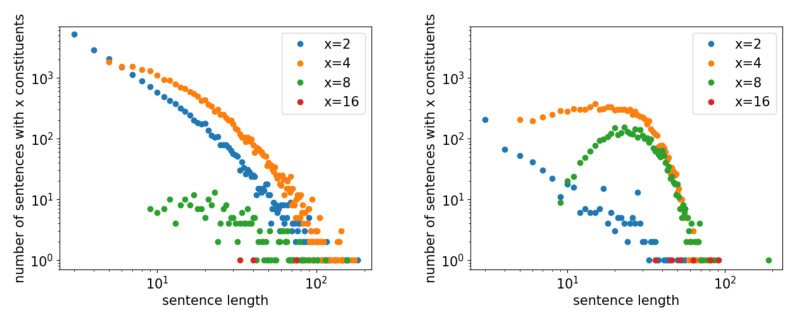
u(n,x), the number of sentences with *x* main constituents, with respect to the sentence length *n*. The left graph is for random sentences, while the right graph is for the PTB.

Figure 7 shows the u(n,x) function for random sentences (left graph) and the PTB (right graph). Each graph shows the distribution u(n,x) with respect to the length *n*, with plots in different colors for different *x* values. In the left graph for random sentences, the *u* function follows a power law, i.e., $u\left(n,x\right)\propto n^{-a}$ up to a certain *x*. The power tendency already shows a convex shape at x=4; however, we may still consider the case of the power distribution for analytical purposes.

When u(n,x) follows a power distribution, both the numerator and the denominator of *v* are power functions. Another factor of *n* is multiplied within the sum of the numerator, v∝x; therefore, for y(x), dividing this *v* by *x* makes *D* independent of *x*, which provides the analytical rationale for the constant D=0 in the left graph of Figure 5. Below, this case of D=0 is referred to as the *baseline*.

Eliminating sentences with length $n<N_{min}$ means that the left part of this left graph is eliminated. When the sentence length is *n* and the number of main constituents is *x*, the size *y* becomes

(4) y=n/x.

By eliminating data points for $n<N_{min}$, small *y* values are eliminated as well. Then, we should observe a bias in which the average *y* becomes larger for small *x*, i.e., $x<N_{min}$. Note that this increase in *y* is larger for a smaller *x*, because all sentences of length $x<n<N_{min}$ are eliminated. For example, when x=5 and $N_{min}=10$, all sentences of length 6, 7, 8, or 9 are eliminated; thus, the number of eliminated sentences is larger for a smaller *x*.

In fact, this elimination process stops at $x=N_{min}$. Therefore, the case of $x>N_{min}$ should not change from the baseline, and indeed, the last five points in the two graphs of Figure 5 are exactly the same. Therefore, *y* shows a decreasing tendency *until* $N_{min}$. Note that the discussion above used $N_{min}=10$ only as an example to show how the elimination process works. If $N_{min}$ is set to a larger number, then the plot will monotonically decay until then.

In other words, the Menzerath property, as defined in Section 2 (i.e., the monotonic decay of *y* with respect to *x* for a certain range of *x*), is reproducible even with random sentences, and therefore, it does not characterize language. As we are interested in what really characterizes language in relation to this phenomenon, we must delve more deeply into the details of what we have seen.

## 6. Discussion

Here, we can conjecture on what characterizes natural language in relation to the Menzerath property.

The lengths of many natural language sentences are much longer than $N_{min}=10$, as can be seen from the first three columns of Table 1. On the other hand, the number of constituents, *x*, is much smaller than that, as discussed above. For random sentences, it was this difference in the ranges of *x* and the sentence lengths, i.e., “no shorter sentences,” that caused the Menzerath property. Given that natural language has a similar difference in the ranges of *x* and sentence lengths, i.e., that it lacks shorter sentences, we cannot completely deny that the Menzerath property of natural language is partly produced by a similar statistical effect.

The true cause, however, could be more complex and is thus deemed to be mixed. Above all, the u(x,n) function for natural language shows a different shape from the shapes for random sentences. For example, consider the shapes for the PTB in the right graph of Figure 7. The graph does not differ much from graphs for other real samples and is thus quite typical. However, it shows entirely different functional shapes, depending on *x*, from the random sentences in the left graph.

For x=2 (blue points), the plot might show a power-like function, indicating the possibility of a random tendency like that shown in the left graph; however, the relative frequency with respect to other *x* values is much lower. For x>2, u(x,n) takes much more interesting functional shapes that show the reality of natural language. In particular, for larger *x*, these plots show how natural language is characterized by having different distributional shapes from those of random sentences. Especially when x=8, for random sentences, the distribution is perhaps similar to some power function, although it almost disappears. For natural language, however, there is a remarkable rise for x=8. This shape is similar to the shape of the distribution for entire sentence lengths when modeled via log-normal or gamma functions [34,35,36]. In other words, this *u* function represents the cause of the Menzerath property of natural language.

We can conjecture why the u(x,n) function has such different shapes from random sentences through reasoning via vocal data, including speech [8] and gelada calls [12]. When we utter one long sentence, the limitation in its duration (for instance, due to the breath length) leads to the tendency to compress a constituent. The data in this article is syntactic and written, and thus, the source of the *limitation* should take a different form; however, orality may influence the written form. We could conjecture that when an overall sentence is complex, having many main constituents, the complexity of every constituent is suppressed, which enables humans to process the structure. This notion of *an appropriate sentence length range* depending on *x* could produce interesting shapes for u(x,n). Clarification of this point will require future work.

From an entirely different perspective, previous studies of the Menzerath property from viewpoints other than syntax could indicate other factors that characterize natural language. Torre et al. [8] indicated how the property would not hold for small *x* in speech, and the authors called this a *reverse regime*. In this article, too, the existence of such a regime has been apparent for natural language text in all the figures. However, the reverse regime cannot be reproduced by random sentences. From this perspective, the functional models of Formulas (1) and (2) do not incorporate this regime, either. In the history of studies of the Menzerath property, the reverse regime has likely not been considered important because the head and tail of the plot usually behave differently. Nevertheless, as emphasized by Torre et al. [8], the reverse regime is deemed a characteristic of natural language.

Overall, the understanding gained in this article is that the Menzerath property is almost reproducible with random sentences and does not necessarily characterize natural language. However, the statistical details of the data revealed the differences of natural language text from random sentences, and further study of those details would lead to a better understanding of natural language.

## 7. Conclusions

This article considered the Menzerath law from the perspective of syntactic structures. The Menzerath property indicates that the sizes of a linguistic construct’s parts, *y*, become smaller when the size of the entire construct, *x*, becomes larger, where *y* follows a power law with respect to *x*. Previously, some papers indicated how the Menzerath property holds for a dependency structure with such a scheme [21,22,23,24], where *x* is the number of constituents modifying the predicate of a sentence, and *y* is the size of a constituent modifying the predicate, in terms of the number of words. Each of those papers considered whether the property holds for a specific language; however, the questions of how well the property holds and what happens when it is examined for random sentences remained open.

Thus, this article first examined the Menzerath property for the Universal Dependency dataset (ver. 2.3, 76 languages and 129 corpora) and the Penn Treebank. The property held reasonably well, at least when the number of main constituents was larger than two. The Menzerath property, originally viewed as the decrease of *y* with respect to *x*, held universally for a certain range of *x*; however, the degree of decrease, as quantified via a power function formulation, depended on the corpus and the experimental setting.

Then, to develop the main point of the article, the Menzerath property was investigated for syntactically random sentences. Following the theory, a decreasing tendency was not observed for the random sentences, but the simple elimination of short sentences caused the property to almost hold, showing that it is reproducible even from random sentences. A detailed analysis of the distribution of the number of sentences for different *x* with respect to sentence lengths, however, suggested that the cause could be more complex and has mixed factors. A further detailed study of the data in this direction would reveal the characteristics of natural language sentences, and this remains as a future work.

## Figures and Tables

**Table 1:** Statistics related to the Menzerath property for the Universal Dependency dataset ver. 2.3 [29] (first to fourth rows), the Penn Treebank [28] (fifth row), and the average of 10 randomly generated samples (last two rows). The first three columns indicate the sentence length populations in terms of the number of sentences, the average sentence length, and the proportion of sentences with fewer than 10 words. The 4th and 5th columns list the overall statistics characterizing the nature of the data. The “decrease ratio” indicates the average proportion of data points among all points in the range of 1<x≤16 for which the mean constituent size of *x* decreased as compared with that of x−1. The **standard deviation** indicates the average standard deviation for the data points. The rest of the columns indicate the estimates for the fit functions. The functional parameters *D* (6th column) in Formula (1) and *B* and *C* (8th and 9th columns) in Formula (2) were estimated using only the range of $x_{min}=3\leq x\leq x_{max}=10$. The points at x=1,2 were not used for the fitting because they often behaved differently for certain corpora. The error values, which were calculated as the standard deviation from the fit function, are listed in the 7th column for Formula (1) and the last column for Formula (2).

Corpus	Number of Sent.	Avr. Len. Sent. (%)	Ratio of Len. < 10	Decrease Ratio	St. Dev.	D	Err	B	C	Err
**Czech-PDT** **len ≥ 10**	87,913 64,137	17.17 21.53	0.27 0.00	0.67 0.80	5.05 6.01	0.38 0.82	1.48 1.28	0.87 1.34	−0.08 −0.13	2.42 0.59
**Russian-SynTagRus**	61,889	17.90	0.23	0.60	5.53	0.19	1.29	1.02	−0.14	2.57
**Japanese-BCCWJ**	57,109	22.30	0.25	0.80	7.50	0.41	1.35	−0.16	0.10	0.50
**Universal Dependency avr./corpus**	7586.6	18.40	0.29	0.76	4.96	0.31 ± 0.21	1.12	0.48	−0.03	1.63
**Penn Tree Bank**	49,208	23.62	0.09	0.73	4.79	0.66	3.25	1.21	−0.09	5.26
**randomized avr.**	100,000.0	11.85	0.59	0.55	6.72	0.00 ± 0.01	0.56	−0.09	0.02	0.56
**len ≥ 10**	40,722.0	22.28	0.00	0.74	7.88	0.71 ± 0.02	1.16	1.18	−0.12	0.62

## Data Availability

Both the Universal Dependency dataset and the Penn Treebank are publicly available.
